# Effect of metformin on the survival of patients with ALL who express high levels of the ABCB1 drug resistance gene

**DOI:** 10.1186/s12967-018-1620-6

**Published:** 2018-09-03

**Authors:** Christian Ramos-Peñafiel, Irma Olarte-Carrillo, Rafael Cerón-Maldonado, Etta Rozen-Fuller, Juan Julio Kassack-Ipiña, Guillermo Meléndez-Mier, Juan Collazo-Jaloma, Adolfo Martínez-Tovar

**Affiliations:** 1 Servicio de Hematología, Hospital General de México, “Dr. Eduardo Liceaga”, Ciudad de México, México; 2 Laboratorio de Biología Molecular, Servicio de Hematología, Hospital General de México, “Dr. Eduardo Liceaga”, Ciudad de México, México; 3 Dirección de Investigación, Hospital General de México, “Dr. Eduardo Liceaga”, Ciudad de México, México

**Keywords:** ATP binding cassette subfamily B member 1 (ABCB1), Quantitative real-time polymerase chain reaction (qRT-PCR), Acute lymphoblastic leukemia (ALL)

## Abstract

**Background:**

In acute lymphoblastic leukemia (ALL), high ABCB1 gene expression has been associated with treatment resistance, which affects patient prognosis. Many preclinical reports and retrospective population studies have shown an anti-cancer effect of metformin. Therefore, the objective of this study was to assess the effect of metformin on the treatment regimen in patients with ALL who exhibited high levels of ABCB1 gene expression and to determine its impact on overall survival.

**Methods:**

A total of 102 patients with ALL were recruited; one group (n = 26) received metformin, and the other received chemotherapy (n = 76). Measurement of ABCB1 transcript expression was performed using qRT-PCR prior to treatment initiation. Survival analysis was performed using Kaplan–Meier curves. The impact of both the type of treatment and the level of expression on the response (remission or relapse) was analyzed by calculating the odds ratio.

**Results:**

The survival of patients with high ABCB1 expression was lower than those with low or absent ABCB1 gene expression (p = 0.030). In the individual analysis, we identified a benefit to adding metformin in the group of patients with high ABCB1 gene expression (p = 0.025). In the metformin user group, the drug acted as a protective factor against both therapeutic failure (odds ratio [OR] 0.07, 95% confidence interval [CI] 0.0037–1.53) and early relapse (OR 0.05, 95% CI 0.0028–1.153).

**Conclusion:**

The combined use of metformin with chemotherapy is effective in patients with elevated levels of ABCB1 gene expression. *Trial registration* NCT 03118128: NCT

## Background

Acute lymphoblastic leukemia (ALL) is the most frequent malignancy in children and adolescents [1, 2] and is characterized by lack of control in the normal mechanisms of proliferation, differentiation and apoptosis inhibition. Despite the great diversity of treatments, cure remains unfavorable in adult patients: the cure rate is almost 90% in children but is unfortunately approximately 30–40% in adults [3, 4]. Therefore, new treatment strategies that are less toxic, accessible and that improve cure rates are needed [5, 6]. The mechanisms of tumor failure in the overexpression of drug transporters involved in the expulsion of drugs, such as daunorubicin, doxorubicin, etoposide, vincristine or vinblastine, and mitoxantrone, prevent the drug’s mechanism of action and limit its activity [7–12] Among the most studied transporters is the ABCB1 gene, which encodes a 170-kDa protein called P-glycoprotein (Pgp-170) [13–15] Our group previously reported that 52% of patients with ALL overexpressed the ABCB1 gene, which was associated with lower survival [16]. Metformin (1,1-dimethylbiguanide hydrochloride) has been shown to have anti-proliferative, anti-invasive, and anti-metastatic effects in multiple cancer cells [17, 18]. At the molecular level, metformin activates metabolic stress via the AMPK pathway, resulting in the inhibition of several metabolic pathways, such as the mitochondrial respiratory chain and the signaling pathway mediated by nuclear factor kappa B (NF-KB) [19–21] This effect could be beneficial in patients with ALL since it has been demonstrated that NF-KB is responsible for activating the overexpression of ABCB1 genes [22–25] The use of metformin in the pre-induction stage combined with steroids may enhance chemosensitivity and promote the survival of patients with ALL.

Preclinical studies have shown that the use of metformin combined with chemotherapeutics such as doxorubicin, paclitaxel and carboplatin have antiproliferative effects in a mouse xenograft model of breast cancer [26]. Additionally, low doses of metformin inhibit the cellular transformation of four genetically different types of breast cancer. The combination of metformin and doxorubicin reduces the tumor mass and prevents relapse [27].

Thus, the objective of the present study was to evaluate the efficacy of metformin in a cohort of patients diagnosed with Philadelphia-negative immunophenotype B ALL at the pre-induction stage expressing different levels of multi-resistance gene (ABCB1) and its treatment response as well as overall patient survival. We demonstrated the protective effect of metformin in the pre-induction stage in patients with high ABCB1 gene expression levels, who exhibited prolonged overall survival and a reduced risk of refractoriness and relapse compared to the control group.

## Methods

### Study design

**Fig. 1:**
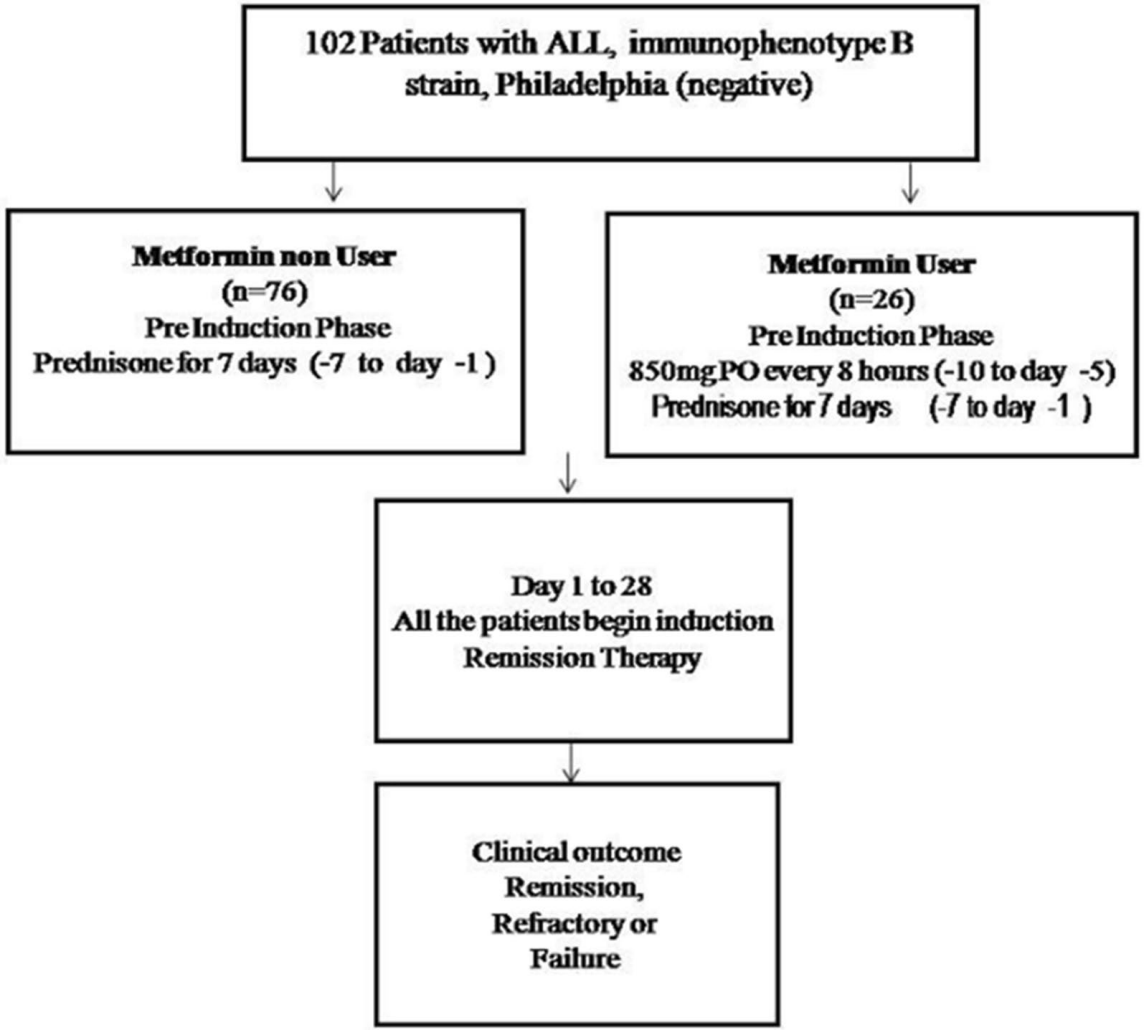
Treatment scheme used in the two ALL patient treatment groups

This was a prospective clinical study with adult ALL patients diagnosed according to the Franco–American–British classification and confirmed by flow cytometry. The assignment of the groups was through simple randomization (3:1). After informed consent was obtained, 102 patients were included in the study; 26 patients received metformin (metformin users) in the pre-induction stage at 850 mg PO every 8 h in conjunction with the chemotherapy regimen LALHGM07, and 76 patients received chemotherapy (metformin non-users) exclusively (Fig. 1).

All patients had a leukemia B phenotype, and patients with diabetes were excluded, as were cases carrying the Philadelphia chromosome or a cytogenetic alteration with poor prognosis. Expression levels of the ABCB1 gene were measured prior to initiation of treatment. This clinical trial was approved by the Ethics, Research and Biosafety Committees of the General Hospital of Mexico “Dr. Eduardo Liceaga” D1/15/103/03/57. The study adhered to the principles of the recent revision to the Declaration of Helsinki. All participants provided signed forms indicating prior consent.

### Analysis of ABCB1 expression

Expression levels of the ABCB1 gene were evaluated prior to treatment initiation in both treatment groups. All samples were collected from the bone marrow, and the mononuclear cells were separated using Ficoll-Hypaque Hypaque (Lymphoprep, Nycomed Pharma AS, density 1.077 g/L). The mononuclear cell phase was separated and suspended in PBS medium and stored at − 70 °C. RNA Isolation was performed using TRIzol^®^ (Invitrogen/Life Technologies). The RNA was stored at − 80 °C until needed.

For cDNA synthesis, 2 μg of RNA, final volume of 20 μL was combined with 200 U of the MMLV RT enzyme (Invitrogen, Carlsbad, CA, USA).

### Real-time polymerase chain reaction (qRT-PCR) analysis

The mRNA expression levels of the ABCB1 (Hs01069047) and glyceraldehyde 3-phosphate dehydrogenase (GAPDH; Hs00985689) genes were measured using TaqMan gene expression assays [16] (Applied Biosystems Foster City, CA, USA). The GAPDH gene was used as an endogenous control, and each sample was analyzed in triplicate.

The relative expression levels were calculated using the 2^−∆∆Ct^ method. The high and low expression cut-off points were determined based on the mean values observed in 99 healthy donors [16].

### Response to treatment

The treatment was checked at day 28 based on bone marrow uptake.

*Complete Remission* was defined as the patient having less than 5% of blasts at the end of induction therapy, *Refractory* patients remained leukemic, and *Therapeutic Failure* was defined as the patient dying during therapy. Patients who had complete remission and who presented an increase in the number of blasts (> 5%) at any time were considered to be in *Relapse*. The consolidation phase consisted of the administration of sequential blocks of chemotherapy, including administration of high doses of methotrexate. At the end of the study, the patients started the maintenance phase by administering weekly mercaptopurine and methotrexate for a duration of 2 years. In case of relapse to bone marrow, the patients received rescue therapy.

### Statistical analysis

Data were collected in MS Excel (Microsoft Corporation, Redmond WA, USA) and were analyzed in SPSS statistical software (version 20 for Windows: IBM, Armonk, NY, USA). A survival analysis was performed using Kaplan–Meier curves. The difference between the two groups was determined using the log-rank test (p = 0.05). Both the type of treatment and the level of expression on response (remission or relapse) were analyzed by calculating the odds ratios.

## Results

A total of 102 patients with B-immunophenotype-free were enrolled and divided into two groups: metformin users (n = 26) and metformin non-users (n = 76).

The mean age for the metformin user group was 31 years (range of 18–61 years), and 13 women and 13 men were included, with a median leucocyte count of 42.1 × 10^3^/mcl (range of 1.2–220 × 10^3^/mcl). Regarding the metformin non-user group, the mean age was 35 years (range 18–78 years). The patients were both female (n = 40, 52.6%) and male (n = 36, 47.4%), with a measured leukocyte count 45.1 × 10^3^/mcl (range 0.4–251 × 10^3^/mcl) (Table 1).

**Table 1:** Frequency of ABCB1 gene expression in patients metformin non user and metformin user

	Metformin non-user, n = 76 (75%)	Metformin user, n = 26 (25%)
Age (median)	35 (18–78)	31 (18–61)
Gender		
Male	36 (47.4)	13 (50)
Female	40 (52.6)	13 (50)
Leukocytes (median) (10^3^/mcl)	45.1 (0.4–251)	42.1 (1.2–220)
Type of risk		
Standard risk	33 (43.4)	18 (69.2)
High risk	43 (56.6)	08 (30.8)
Expression ABCB1 (%)		
Absence expression	28 (36.8)	8 (30.8)
Low expression	14 (18.4)	6 (23.1)
High expression	34 (44.7)	12 (46.2)
Expression combination (%)		
Low and missing expression	42 (55.3)	14 (53.8)
High expression	34 (44.7)	12 (46.2)
Treatment failure		
Remission complete	47 (61.8)	18 (69.2)
Death or refractoriness	29 (38.2)	08 (30.8)

When analyzing the relative ABCB1 gene expression levels prior to treatment initiation in the 102 patients, 45.1% (46/102) had high expression levels, 19.6% (20/102) had low levels, and only 35.2% (36/102) did not express the gene. The proportions of patients with high expression were similar in the two treatment groups 46.2% (12/26) in the metformin user group and 44.7% (34/76) in the metformin non-user group), as were the low and negative expression levels (Table 1).

**Fig. 2:**
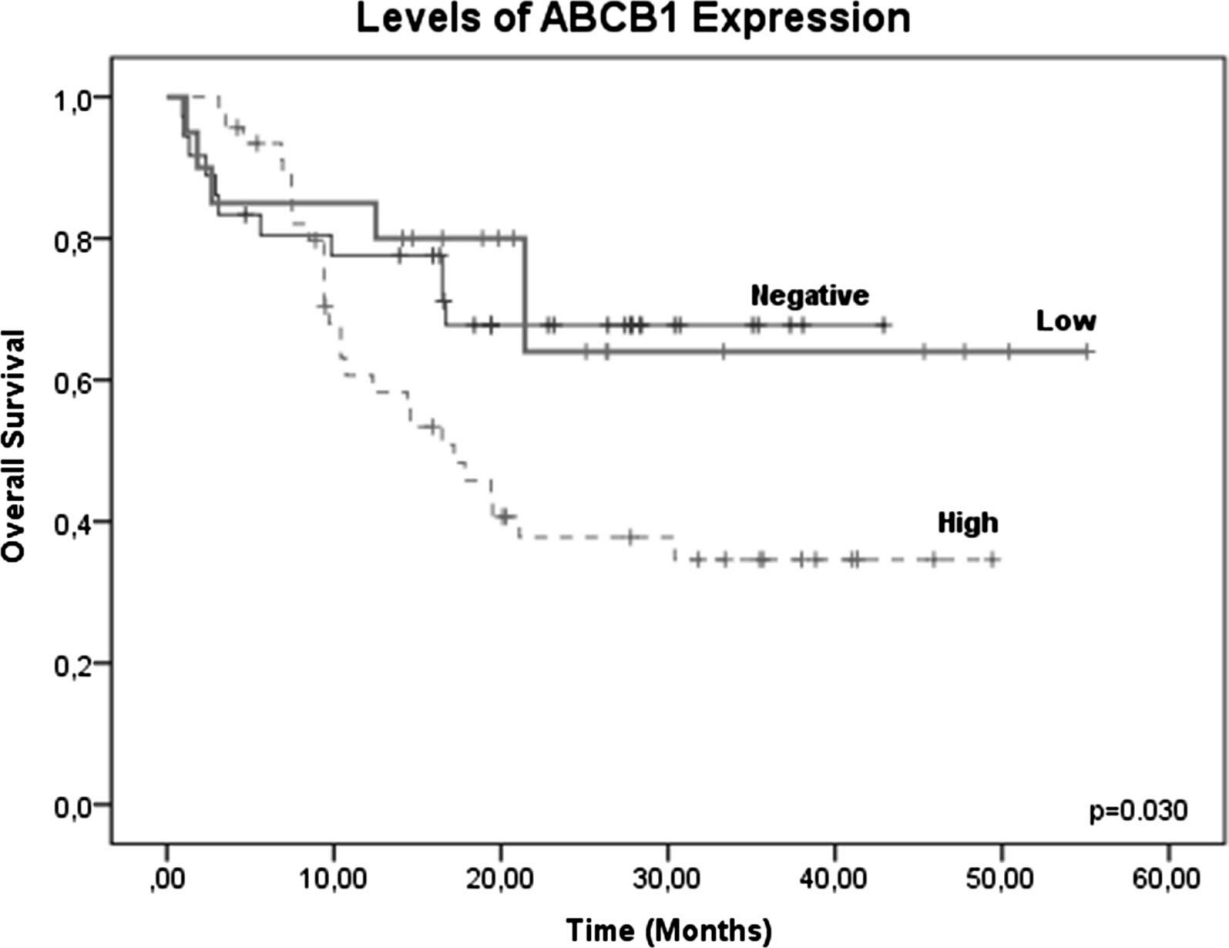
Global survival (OS) in patients with ALL expressing the ABCB1 gene. OS at 60 months in patients expressing the ABCB1 gene was analyzed. Patients with high levels of ABCB1 gene expression had an OS of 41.5% (19/46) in patients with ALL; patients with low or negative ABCB1 expression had OS values of 70% (14/20) and 69.5% (25/36), respectively (p ≤ 0.030, log-rank test)

The effect of expression levels of the ABCB1 gene on overall survival (OS) at 60 months demonstrated that high levels of ABCB1 gene expression were associated with a lower OS of 41.5% (19/46) in ALL patients; low and negative expression had OS values of 70% (14/20) and 69.5% (25/36), respectively. The results indicated a significant decrease in patients with high levels of ABCB1 gene expression, similar to our previously reported findings (p ≤ , log-rank test) (Fig. 2).

**Fig. 3:**
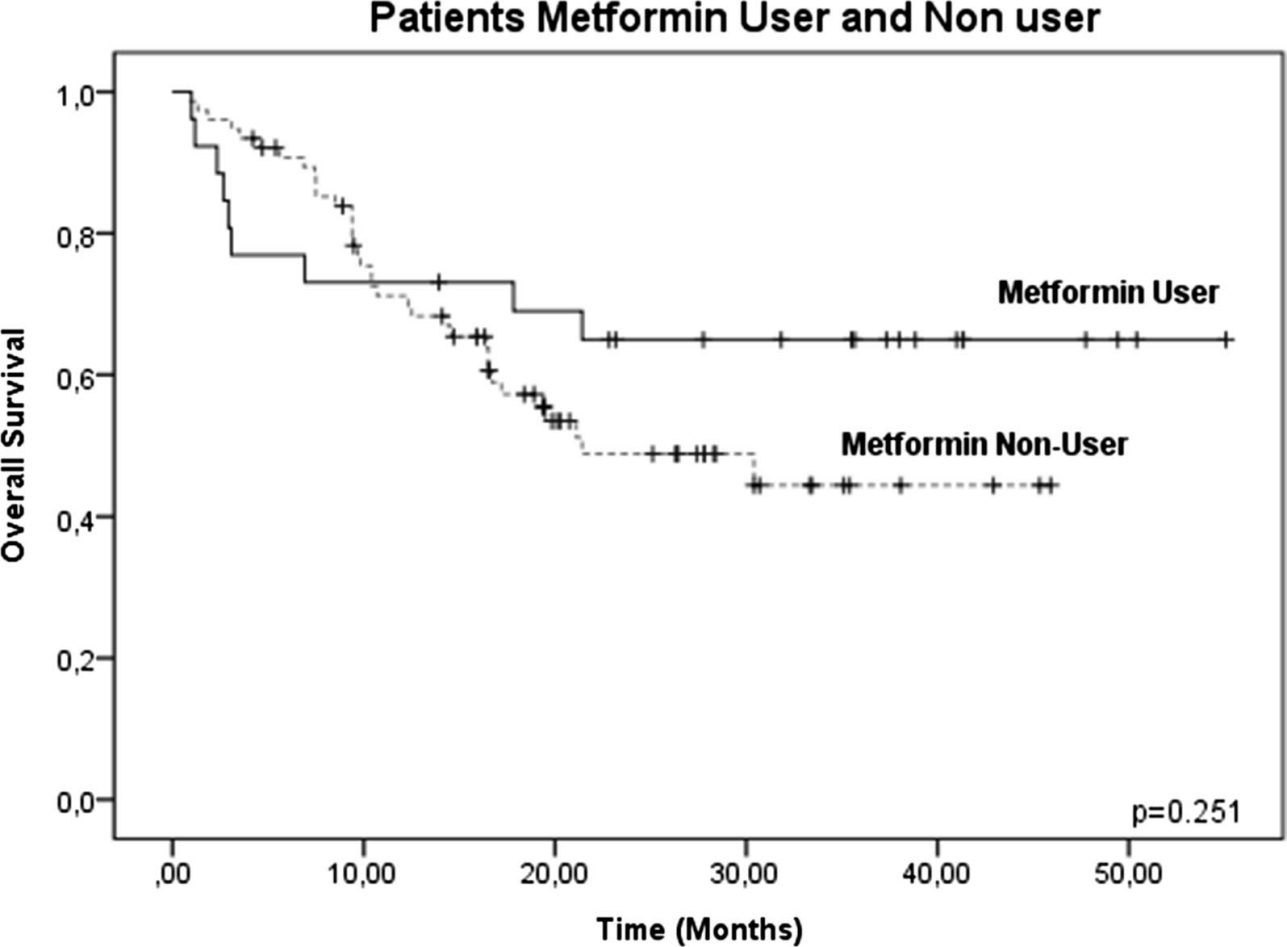
Global survival (OS) in patients with ALL treated with metformin (metformin users and metformin non-users). OS at 60 months in patients treated with metformin was analyzed, and no significant differences were found between the two groups (p = 0.251, 95% CI)

**Fig. 4:**
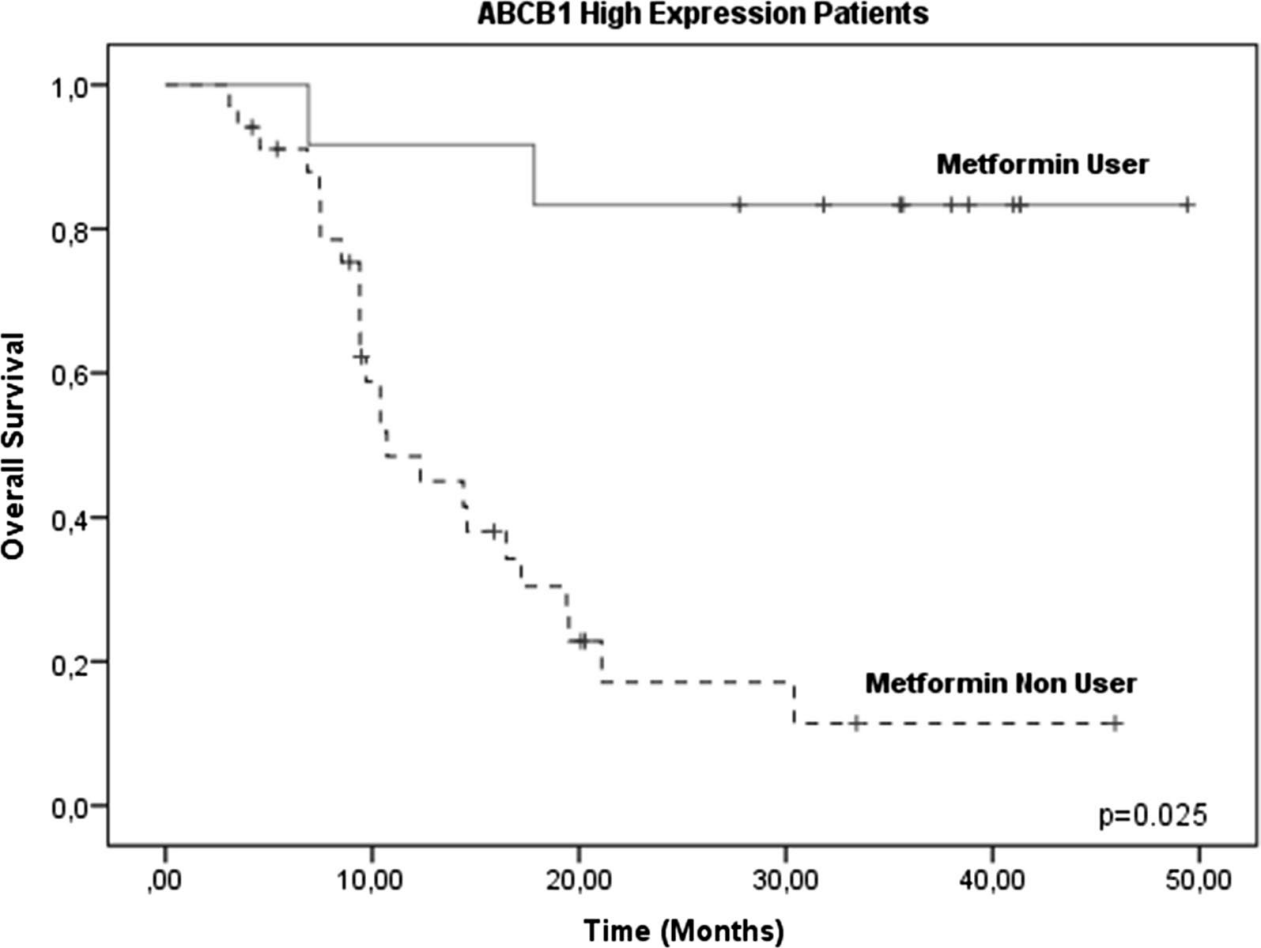
Global survival (OS) in patients with metformin-treated ALL (metformin users) and ALL without metformin treatment (metformin non-users) with elevated levels of ABCB1 gene expression. OS at 60 months in Metformin-treated patients and high levels of ABCB1 expression was analyzed, and metformin had a protective survival benefit (p = 0.025, 95% CI)

**Fig. 5:**
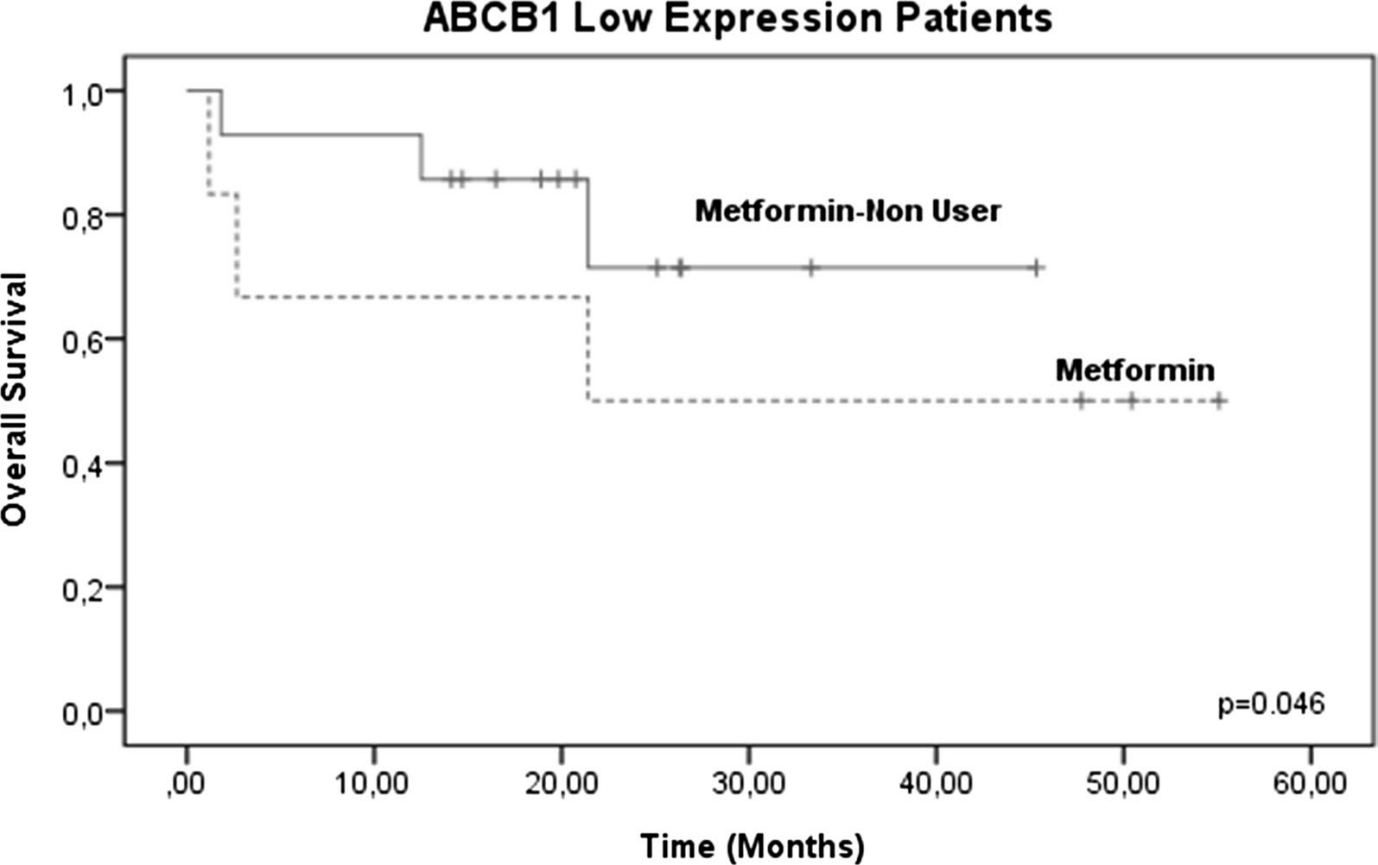
Global survival (OS) in patients with metformin-treated ALL (metformin users) and ALL without metformin treatment (metformin non-users) with low ABCB1 gene expression levels. No significant changes were observed in OS in the patients with low ABCB1 gene expression levels (p = 0.046, 95% CI)

**Fig. 6:**
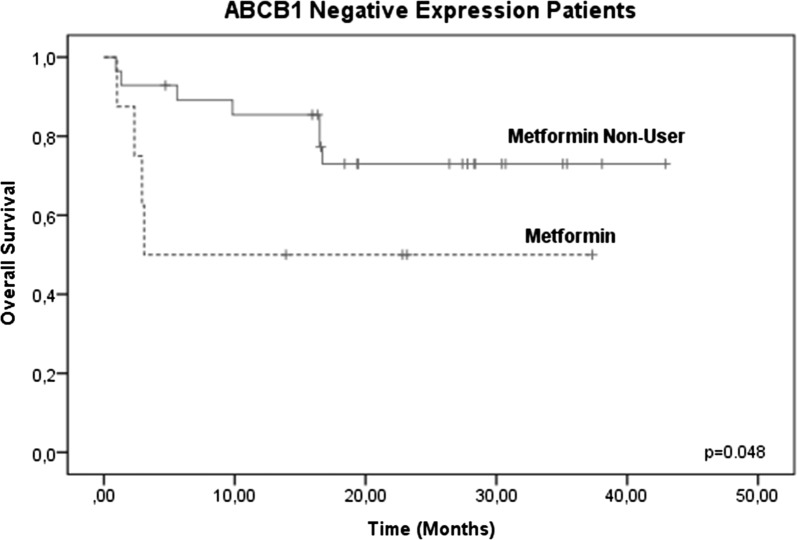
Global survival (OS) in patients with metformin-treated ALL (metformin users) and ALL without metformin treatment (metformin non-users) with negative ABCB1 gene expression levels. No significant changes were observed in OS in the patients with low ABCB1 gene expression levels (p = 0.048, 95% CI)

However, when evaluating the two treatment groups (metformin users and metformin non-users), there were no significant differences between the groups (p = 0.251, 95% confidence interval [CI]) (Fig. 3), confirming that no therapeutic benefit was evident in the OS in 102 patients, without considering ABCB1 gene expression levels. Therefore, we analyzed only those patients who reported high levels of ABCB1 gene expression (46/102) and its effect on OS. In these patients, increased survival was observed in the metformin user group (83.3%, 10/12) compared with the metformin non-user group 26.6%, (9/34), decreasing the risk of therapeutic failure (p = 0.025, 95% CI) (Fig. 4). No significant changes were observed in OSB patients with low or negative levels of ABCB1 gene expression (Figs. 5, 6).

When assessing the type of risk in the treatment groups, in terms of refractoriness, ABCB1 gene expression demonstrated an increased risk of drug failure (odds ratio [OR] 7.48, 95% CI 0.36–153.79) with other clinical variables, such as age over 35 years (OR 2.25, 95% CI 0.30–16.63) and white blood cell count (OR 6.00, 95% CI 0.56–63.98). Regarding relapse, high levels of ABCB1 gene expression were also associated with an increased risk of relapse (OR 2.91, 95% CI 0.28–30.29) compared with clinical variables such as age greater than 35 years (OR 3.42, 95% CI 0.49–23.77) and leukocyte count (OR 8.57, 95% CI 0.82–89.04). In the metformin user group, metformin could function as a protective factor against both therapeutic failure and early relapse (OR 0.07, 95% CI 0.003–1.53 and OR 0.05, 95% CI 0.0028–1.15) (Table 2).

**Table 2:** ALL patients mortality and OR model according to use of metformin

	Refractoriness			Relapse		
	OR	(95% CI)	p	OR	(95% CI)	p
Age > 35 years	*2.25*	0.30–16.63	0.426	*3.42*	0.49–23.77	0.214
Leukocytes > 30× 10^3^/mcl	*6.00*	0.56–63.98	0.137	*8.57*	0.82–89.04	0.072
Metformin user	*0.07*	0–1.5	0.091	*0.05*	0–1.15	0.061
Expression ABCB1 negative or high	*7.48*	0.36–153.79	0.192	*2.91*	0.28–30.29	0.896

*OR* odds ratio, *CI* confidence interval, *ABCB1* ATP binding cassette subfamily B member 1

## Discussion

Although most of the clinical evidence was obtained from the observational, studies or patients with advanced stages of treatment, it can be concluded that metformin is beneficial for the prevention of the appearance of new cancers and as an adjuvant to different treatment modalities [26–29]. These results indicate that the capacity of metformin to be combined with different treatment strategies is very broad; its application is more relevant for tumors that express AMPK/mTOR activity [30]. It can also be applied with drugs involved in cellular metabolism or in the synthesis, where effectiveness is improved with new strategies such as immunotherapy [25]. Recently, the use of metformin has been combined with tyrosine kinase inhibitors (imatinib, sorafenib), and it has been effective in improving responses in cases of not only chronic myeloid leukemia but also myeloid leukemia with mutations in FLT3/ITD [28, 29]. Regarding the association between high ABCB1 gene expression and the activation of energy-dependent P (gp170) glycoprotein (ATP), energy depletion is an attractive strategy to overcome resistance to different drugs, including cytotoxic drugs (daunorubicin, doxorubicin, etoposide, vincristine or vinblastine, and mitoxantrone) or even antibiotics [10, 31–34]. To date, there have been few trials to explain the effects of metformin on the expression of drug resistance genes. Recently, in a cell model of hepatocarcinoma cells (HepG2, HepG2/ADM, LO2), Wu et al. reported that the addition of metformin (1 μmol/L for 24 h and subsequently for 48 h at 1.5 μmol/L) decreased gp170 expression by blocking the expression of nuclear factor NF-kB, inducing hepatocyte apoptosis. Similar to the model of Kim and collaborators in the doxorubicin-resistant MCF-7/Dox cell line, Xue and colleagues were also able to demonstrate the effects of metformin on the decrease in drug resistance in association with 2-deoxyglucose by decreased glucose uptake and ATP depletion by increasing the toxic effect of doxorubicin by compromising the function of the p53 tumor suppressor gene [35, 36]. These results are greatly relevant, since metformin, which is an expensive and accessible drug with a tolerable safety profile (rare cases of lactic acidosis), may ameliorate one of the main therapeutic failure mechanisms in cancer [37, 38]. Although there is strong evidence from in vitro trials, perhaps the major criticism of the use of metformin is that most of the evidence is obtained from observational trials [39–41], and evaluating its effects in a few randomized placebo trials have failed to identify a beneficial effect of treatment [42–46].

In our study, the pretreatment baseline ABCB1 gene expression was defined, which allowed for an independent stratification of clinical risk. According to the previous results, patients with high levels of ABCB1 gene expression have an adverse prognosis [16]. However, when metformin was added to the pre-induction stage for this group of patients, its beneficial effect manifested as improved clinical outcomes and a reduced number of therapeutic failures. This finding was similar to the findings of some observational studies or case reports in which the effect of metformin on the treatment of tumors in advanced or metastatic cases was considered beneficial [47–51].

## Conclusion

Although clinical evidence in leukemia patients is scarce, we consider that its use in this stage of treatment is beneficial only in patients with high levels of ABCB1 gene expression. Therefore, a greater number of studies in this specific subgroup of patients should be performed to determine whether that benefit could be extended for the detection of minimal residual disease or in patients after transplantation of hematopoietic progenitor cells.

## Abbreviations

Ph^+^ALL: Philadelphia chromosome-positive acute lymphoblastic leukemia
qRT-PCR: real-time quantitative polymerase chain reaction
BCR-ABL: breakpoint cluster region, Abelson
TKIs: tyrosine kinase inhibitors
BM: bone marrow
B-ALL: B cell acute lymphoblastic leukemia
WHO: World Health Organization
BMMNCs: bone marrow mononuclear cells
MoAbs: monoclonal antibodies
Ara-C: cytarabine arabinoside
dNTP: deoxy-ribonucleotide triphosphate
CR: complete remission
RFS: relapse-free survival
OS: overall survival
FAB: French-Britain-American
WBC: white blood cell
OR: odds ratio
HR: hazard ratio
CI: confidence interval
